# Alterations in the mutagenicity and mutation spectrum induced by benzo[a]pyrene instilled in the lungs of *gpt* delta mice of various ages

**DOI:** 10.1186/s41021-015-0004-x

**Published:** 2015-06-16

**Authors:** Yasunobu Aoki, Akiko H. Hashimoto, Yoshiki Sugawara, Kyoko Hiyoshi-Arai, Sataro Goto, Kenichi Masumura, Takehiko Nohmi

**Affiliations:** National Institute for Environmental Studies, Center for Environmental Risk Research, 16-2 Onogawa, 305-8506 Tsukuba, Ibaraki Japan; Juntendo University, Graduate School of Health and Sports Science, 270-1695 Inzai, Chiba Japan; Division of Genetics and Mutagenesis, National Institute of Health Sciences, 158-8501 Setagaya-ku, Tokyo Japan; Present address: University of Shizuoka, School of Nursing, 422-8526 Suruga-ku, Shizuoka Japan

**Keywords:** Aging, Air pollutant, *In vivo* mutation, Oxidative stress, Transgenic rodent assay

## Abstract

**Introduction:**

To examine whether the mutagenic potential of lung exposure to air-borne environmental mutagens is age dependent, we administered 1 mg of benzo[a]pyrene intratracheally to 11- and 24-month old (middle-aged and old, respectively) *gpt* delta transgenic mice that harbor *gpt* (guanine phosphoribosyltransferase) genes integrated in the genomic DNA as a target for mutation detection, and then analyzed the benzo[a]pyrene-induced and spontaneous *in vivo* mutations and mutation spectrum in the lungs.

**Results:**

The mutant frequencies in the lungs of the 11- and 24-month–old control (vehicle-treated) *gpt* delta mice were 1.14 ± 0.22 × 10^−5^ and 1.00 ± 0.20 × 10^−5^, respectively, which are significantly higher than that observed for the control 3-month–old (young) mice (0.59 ± 0.13 × 10^−5^) in our previous studies, indicating that spontaneous mutation in the lung increases with age. The mutant frequencies in 11- and 24-month–old mice treated with benzo [a] pyrene were 1.5- and 2.3-fold, respectively, that of the age-matched control mice, and 4.3-fold that of the 3-month–old mice in our previous studies. Analysis of mutation spectra showed that both G:C to A:T transitions and G:C to T:A transversions were predominant in the lungs of control mice at all ages. In benzo [a] pyrene-treated mice in our previous studies, G:C to T:A transversions were the predominant type of mutation (55 %) at 3 months. Here we found that their frequency was dramatically reduced to 18 % by 24 months, and the G:C to A:T transitions became the predominant type of mutation in 24-month–old mice (41 % [16 % at CpG sites]).

**Conclusions:**

Our findings suggest that susceptibility to benzo[a]pyrene is highest in young mice and is elevated again in old age. The elevation of G:C to A:T transitions was observed following benzo [a] pyrene administration in the lungs of aged mice, and accelerated cytidine deamination is speculated to contribute to this elevation.

## Introduction

Accumulation of mutations in the genome is considered to be at least in part responsible for the phenomenon of aging. The increase in genomic mutation frequency with age is believed to be a factor in the age-related functional decline of homeostasis and resistance to stressors, which leads to diseases such as cancer [1]. However, it remains to be clarified how vulnerable groups, such as young and old individuals, are susceptible to environmental mutagens and related environmental stressors. For instance, young (3-month–old) rats are more susceptible than adult (11-month–old) rats to acrylamide-induced testicular genotoxicity [2].

Transgenic rodents that harbor exogenous genes integrated in the genomic DNA, as a target for mutation detection, are a useful system for the study of *in vivo* somatic mutations caused by environmental mutagens. The widely used transgenic lines, Muta mouse, Big Blue rodents, and *gpt* delta rodents, harbor the *Escherichia coli* genes, *lacZ* (β-galactosidase), *lacI* (lac repressor), and *gpt* (guanine phosphoribosyltransferase), respectively [3]. Use of these model animals to evaluate the level of mutations accumulated in aged animals has revealed that the mutation frequency increases spontaneously with age in various organs including liver, spleen, small intestine, kidney, and heart [4–10], but the magnitude of the increase is different among the organs.

From the viewpoint of interactions between the genome and the environment, the lung is a unique organ. In the lung, air-borne environmental mutagens directly contact the pulmonary epithelial cells and induce mutations in the genomic DNA, whereas mutagens reach most organs via the blood circulation system. Therefore, here we chose to use the lung to address how the susceptibility to environmental mutagens differs among age groups. We selected to use benzo[a]pyrene (B[a]P), because it is a common air-borne mutagen generated by the burning of fossil fuels. Previously, we reported that B[a]P (0.5–2 mg per animal) induces a linear dose-dependent increase in mutation frequency in the lungs of 3-month–old *gpt* delta mice following a single intratracheal instillation [11]. Here, to determine whether the magnitude of the elevation of *in vivo* mutant frequency following treatment with an environmental mutagen is age dependent, we examined the mutant frequency and types of mutations in the *gpt* gene in B[a]P-instilled lungs of 11- and 24-month–old *gpt* delta mice (representative of middle-aged, and old animals, respectively), comparing with those in 3-month–old *gpt* delta mice (representative of young animals).

## Materials and methods

### Treatment of mice

Male *gpt* delta mice (9-weeks old; body weight, ~25 g), which carry approximately 80 copies of lambda EG10 DNA on each chromosome 17 on a C57BL/6 J background [12], were obtained from Japan SLC (Shizuoka, Japan). The mice were maintained at 24 to 26 °C with 55 % to 75 % humidity and a 12-h light–dark cycle, and were fed CA-1 diet (Japan Clea Co., Tokyo, Japan) with water *ad libitum*, in the specific-pathogen–free (SPF) animal facility of the National Institute for Environmental Studies. The animals were anesthetized with 4 % halothane (Hoechst Japan, Tokyo, Japan) in a desiccator until they did not respond to a tactile stimulus. A single dose of B[a]P (1 mg, Wako Pure Chemical Industries, Osaka, Japan) dissolved in 50 μL of tricaprylin (Sigma-Aldrich, St. Louis, MO, USA) was intratracheally instilled via a polyethylene tube [11, 13]. Control mice were treated with 50 μL of vehicle (tricaprylin) alone; this dose of B[a]P is within the range (0.5–2 mg) that causes a linear dose-dependent increase in mutant frequency in the lungs of 3-month–old *gpt* delta mice [11]. The mice were sacrificed 14 days after the administration, and their lungs were removed, frozen in liquid nitrogen, and stored at −80 °C until the DNA was isolated. The animal studies were approved by the Animal Care and Use Committee of National Institute for Environmental Studies.

### DNA isolation and *in vitro* packaging of DNA

High-molecular-weight genomic DNA was extracted from the lungs by using the RecoverEase DNA Isolation Kit (Agilent Technologies, Santa Clara, CA, USA). Lambda EG10 phages containing the *gpt* gene were recovered from the genomic DNA by using Transpack Packaging Extract (Agilent Technologies).

### Mutation assay and DNA sequencing analysis of the *gpt* gene in 6-TG-resistant colonies

The *gpt* mutagenesis assay was performed according to previously described methods [14]. To convert the phage DNA into plasmids, *E. coli* strain YG6020 expressing Cre recombinase was infected with the rescued phage. The bacteria were then spread onto M9 salts plates containing chloramphenicol (Cm) and 6-thioguanine (6-TG) [14], and incubated for 72 h at 37 °C for selection of the colonies harboring a plasmid carrying the chloramphenicol acetyltransferase (*cat*) gene and a mutated *gpt* gene. The 6-TG-resistant colonies were streaked onto selection plates for confirmation of the resistant phenotype. The cells were then cultured in LB broth containing 25 mg/mL Cm at 37 °C and collected by centrifugation. The bacterial pellets were stored at −80 °C until DNA sequencing analysis was performed. Mutant frequencies for the *gpt* gene were calculated by dividing the number of colonies growing on M9 + Cm + 6-TG agar plates by the number of colonies growing on M9 + Cm agar plates. PCR and DNA sequencing analysis of 6-TG-resistant mutants were performed as previously reported [11].

### Statistical analysis

All data are expressed as means ± SD. Statistical significance was evaluated by using Student’s *t*-tests. A statistical analysis of mutational spectra was performed by using the Adams-Skopek test [15, 16] and Chi-square test. *P* values less than 0.05 were considered to be statistically significant.

## Results and discussion

### Mutant frequencies in the lungs of B[a]P-treated *gpt* mice

To examine which age group is most susceptible to a common air-borne environmental mutagen, the mutant frequency and the mutation spectrum in the lungs of 11- and 24-month–old *gpt* delta mice (representative of middle-aged and old animals, respectively) following treatment with B[a]P and age-matched controls (vehicle-treated) were analyzed and compared with our published data for 3-month–old mice (representative of young animals) [11, 17, 18].

The mutant frequencies in the lungs of 11- and 24-month–old control (vehicle-treated) mice were 1.14 ± 0.22 × 10^−5^ and 1.00 ± 0.20 × 10^−5^, respectively. In contrast, the mutant frequency in the lungs of 3-month–old control mice was 0.59 ± 0.13 × 10^−5^ according to the combined data from our previous reports [11, 17, 18]. Consistent with previously reported observations in various organs such as liver and spleen [10, 19, 20], these observations indicate that the frequency of spontaneous mutants in the lung increased with age.

A single administration of 1 mg of B[a]P elevated the mutant frequency in 11- and 24-month–old mice to 1.68 ± 0.19 × 10^−5^ and 2.25 ± 0.54 × 10^−5^, respectively, which was 1.5- and 2.3-fold the mutant frequency in the respective age-matched control mice (Table 1). We previously reported that B[a]P instillation to the lungs of 3-month–old mice elevated the mutant frequency to 2.52 ± 0.33 × 10^−5^ [11] which was 4.3-fold the frequency observed in the 3-month–old control mice in our previous studies [11, 17, 18].

**Table 1:** Mutant frequencies in B[a]P-instilled and control lung of *gpt* delta mice

B[a]P	Time	ID of animals	Number of colonies		Mutant frequency	Average mutant frequency ± SD
(mg)	(months)		Mutant	Total	(×10^−5^)	(×10^−5^)
0	3	1^a^	5	800,800	0.62	
		2^a^	5	1,113,600	0.45	
		3^a^	5	702,400	0.71	
		4^b^	3	441,600	0.68	
		5^b^	3	643,200	0.47	
		6^b^	3	828,000	0.36	
		7^c^	7	1,016,000	0.69	
		8^c^	6	836,800	0.72	
		9^c^	3	524,200	0.57	
		Total	40	6,906,600		0.59 ± 0.13
	11	1	3	311,000	0.96	
		2	5	530,000	0.94	
		3	30	2,389,000	1.26	
		4	28	1,915,000	1.46	
		5	34	3,117,000	1.09	
		Total	100	8,262,000		1.14 ± 0.22^††^
	24	1	12	1,408,000	0.85	
		2	18	1,464,000	1.23	
		3	14	1,548,000	0.90	
		Total	44	4,420,000		1.00 ± 0.20^†^
ᅟ						
1	3	1 ^a^	11	499,200	2.20	
		2 ^a^	14	556,800	2.51	
		3 ^a^	35	1,225,600	2.86	
		Total	60	2,281,600		2.52 ± 0.33**
	11	1	11	750,000	1.47	
		2	13	700,000	1.86	
		3	14	738,000	1.90	
		4	17	1,061,000	1.60	
		5	27	1,728,000	1.56	
		Total	82	4,977,000		1.68 ± 0.19*^,††^
	24	1	13	456,000	2.85	
		2	36	1,996,000	1.80	
		3	17	809,000	2.10	
		Total	66	3,261,000		2.25 ± 0.54*^,^

*P < 0.01, **P < 0.001 for comparison between B[*a*]P-instilled mice and age-matched control mice

^†^ *P* < 0.01, ^††^ *P* < 0.001 for comparison to equivalently treated 3-month–old mice

^a, b and c^Data from our previous studies ([11, 17, 18], respectively)

Our observations indicate that the order of the age groups in terms of highest to lowest fold of increase in mutant frequency in the lungs following instillation of B[a]P was 3-, 24-, and 11-month–old mice. These results suggest that young mice are the age-group most susceptible to B[a]P, which may be explained age-related changes of the mutant frequency by their relatively high level of DNA replicating activity and cell turnover rate [21] and/or perhaps higher levels of metabolic activation of B[a]P, as well as DNA repair activity, which promote the formation of B[a]P-DNA adducts, and that the susceptibility is elevated again in old age by several possible mechanisms, as discussed later. Age-dependent alteration in the metabolic activation of B[a]P has not been well-documented in the lung, but the activity of cytochrome 1A, a mono-oxygenase that catalyzes the metabolic activation of B[a]P, has been suggested to decline with age in the rat liver [22].

### Characteristics of the *gpt* mutation spectrum

To analyze the age-dependent alterations in the mutation spectra of B[a]P-instilled and control lungs, we sequenced *gpt* mutants recovered from the lungs as shown in Table 2. We observed a significant difference between the mutation spectrum of the 24-month–old control mice studied here and that of the 3-month–old control mice we studied previously [11, 17, 18] (*P* < 0.05, Adams-Skopec test). In our published data from 3-month–old control mice, the most predominant type of base substitution was G:C to A:T transitions (45 % of total mutants), and a half of these transitions were induced at CpG sites (18 % of total mutants), while G:C to T:A (21 %) and G:C to C:G (16 %) transversions were also major base substitutions. Here, were found that the percentages of G:C to C:G transversions among total mutants were less in 11- and 24-month–old control mice than in 3-month–old control mice, but G:C to A:T transitions and G:C to T:A transversions were still predominant (35 % and 14 % of total mutants, respectively) in 24-month–old control mice (Table 2). In contrast, increased proportions of A:T to T:A transversions, A:T to C:G transversions, and base deletions were observed in the lungs of 24-month–old control mice compared with 3-month–old control mice. An increase in base substitutions at A:T has previously been reported in the intestine and spleen of 32-month–old *lacZ* plasmid transgenic mice, and DNA polymerase η was speculated to act as an A:T mutator in the spleen of aged animals [23]. Taken together, these results suggest that an increase in point mutations at A:T may be a common phenomenon in proliferative aged tissues.

**Table 2:** Classification of *gpt* mutations from the lung of B[a]P-instilled and control mice

Type of mutation in the *gpt* gene	Control		B[a]P		Control (months)						B[a]P (months)					
	All ages		All ages		3^a^		11		24		3^b^		11		24	
	Number	%	Number	%	Number	%	Number	%	Number	%	Number	%	Number	%	Number	%
Base substitution																
Transition																
G:C → A:T	68	39	44	26	17	45	36	39	15	35	4	10	20	25	20	41
(at CpG site)	(28)	(16)	(20)	(12)	(7)	(18)	(14)	(15)	(7)	(16)	(1)	(2)	(11)	(13)	(8)	(16)
A:T → G:C	14	8	1	1	3	8	10	11	1	2	0	0	0	0	1	2
Transversion																
G:C → T:A	29	17	51	30	8	21	15	16	6	14	23	55	19	24	9	18
(at CpG site)	(6)	(3)	(33)	(19)	(3)	(8)	(3)	(3)	(1)	(2)	(19)	(45)	(8)	(10)	(6)	(12)
G:C → C:G	12	7	22	13	6	16	4	4	2	5	7	17	12	15	3	6
A:T → T:A	12	7	7	4	1	3	6	7	5	12	0	0	4	5	3	6
A:T → C:G	7	4	3	2	0	0	4	4	3	7	0	0	0	0	3	6
Deletion																
−1	18	10	22	13	3	8	9	10	6	14	4	10	14	18	4	8
>2	6	3	5	3	0	0	2	2	4	9	0	0	2	3	3	6
Insertion	6	3	11	6	0	0	5	5	1	2	2	5	6	8	3	6
Other	1	1	4	2	0	0	1	1	0	0	2	5	2	3	0	0
Total	173	100	170	100	38	100	92	100	43	100	42	100	79	100	49	100

^a^Combined data of our previous studies [11, 17, 18]

^b^Our previous data [11]

We observed that there was a significant difference between the mutation spectra for both 3- and 11-month–old B[a]P-instilled mice (*P* < 0.05, Adams-Skopec test), and 3- and 24-month–old B[a]P-instilled mice (*P* < 0.01, Adams-Skopec test). In B[a]P-instilled groups, we reported previously that G:C to T:A transversions, a major base substitution induced by B[a]P administration, were the predominant type of mutation (55 %) in the lungs of 3-month–old mice in our previous study [11], but surprisingly in the aged mice, the percentage of these mutations was dramatically lowered to be 24 % and 18 % in 11- and 24-month–old mice, respectively (Table 2). In contrast, the percentage of G:C to A:T transitions was observed to increase in B[a]P-instilled lungs age-dependently, and they became the predominant type of mutation in 24-month–old mice (41 % [16 % at CpG sites]). As observed for control lungs, the percentage of G:C to C:G transversions in B[a]P-instilled lungs decreased with age.

The positions of spontaneous and B[a]P-induced *gpt* mutations are listed in Table 3. Among the mutated sequences isolated from the control mice, 6 *gpt* mutations (G:C to A:T transitions) at nucleotides 64, 86, 115, and 406 in 11-month–old mice, at nucleotide 110 in 4 and 24-month–old mice, and at nucleotide 417 in 24-month–old mice, were each observed in three or more mice. These positions therefore are potential hotspots, while G:C to A:T transitions at nucleotides 64 and 110 were reported to be sites of spontaneous mutation in *gpt* delta mice [24]. The G:C to A:T transitions at nucleotides 64 and 110 and the G:C to C:G transversion at nucleotide 340 were also hotspots in 11-month–old B[a]P-instilled mice. Regarding G:C to T:A transversions, we previously observed that nucleotides 115, 140, 143, 189, and 413 were hotspots for G:C to T:A transversions in B[a]P-instilled lungs of 3-month–old *gpt* delta mice [11], but these nucleotides were not hotspots for this base substitution in 11- and 24-month–old mice; rather the hotspots were nucleotides 402 and 406 in B[a]P-instilled lungs of 11-month–old mice, but there was no hotspot in 24-month–old mice.

**Table 3:** DNA sequence analysis of *gpt* mutations obtained from the lung of B[a]P-instilled and control mice

Type of mutation	Nucleo-tide #	Sequence		Change		Amino acid change			Number					
									Control			B[a]P		
									Months			Months		
									3^d^	11	24	3^e^	11	24
Base substitution														
Transition														
G:C → A:T	26	tGg	→	tAg		Trp	→	Stop			1		2^a^	1
	27	tgG	→	tgA		Trp	→	Stop			1		1	
	37	Cag	→	Tag		Gln	→	Stop			1		2	1
	58	Gca	→	Aca	CpG	Ala	→	Thr			1			
	64	Cga	→	Tga	CpG	Arg	→	Stop	1	5^b^	1		5^b^	1
	86	tGg	→	tAg		Trp	→	Stop		3^b^		1		
	87	tgG	→	tgA		Trp	→	Stop		2^a^				
	92	gGc	→	gAc		Gly	→	Asp		2^a^				
	110	cGt	→	cAt	CpG	Arg	→	His	5^c^	4	3^b^		4^b^	6^a^
	115	Ggt	→	Agt	CpG	Gly	→	Ser	1	5^b^	2	1	2^a^	1
	116	gGt	→	gAt		Gly	→	Asp	2	2^a^				1
	128	gGt	→	gAt		Gly	→	Asp	1	2				2
	176	tGt	→	tAt		Cys	→	Tyr						1
	262	Gat	→	Aat		Asp	→	Asn	1					
	274	Gat	→	Aat		Asp	→	Asn	1		1		1	
	281	gGt	→	gAt		Gly	→	Asp						1
	284	gGt	→	gAt		Gly	→	Asp						1
	367	Gat	→	Aat		Asp	→	Asn					1	
	401	tGg	→	tAg		Trp	→	Stop	2^a^	4^a^				1
	402	tgG	→	tgA		Trp	→	Stop				1		1
	406	Gaa	→	Aaa		Glu	→	Lys		3^b^				
	416	tGg	→	tAg		Trp	→	Stop		1				
	417	tgG	→	tgA		Trp	→	Stop	1	1	4^b^			
	418	Gat	→	Aat		Asp	→	Asn	2^a^	2^a^		1	2^a^	2^a^
A:T → G:C	25	Tgg	→	Cgg		Trp	→	Arg	1					
	41	aTc	→	aCc		Ile	→	Thr	1					
	56	cTc	→	cCc		Leu	→	Pro	1	8^a^	1			
	149	cTg	→	cCg		Leu	→	Pro		1				1
	415	Tgg	→	Cgg		Trp	→	Arg		1				
Transversion														
G:C → T:A	3	atG	→	atT		Met	→	Ile		1				
	7	Gaa	→	Taa	CpG	Glu	→	Stop		1				
	15	taC	→	taA		Tyr	→	Stop		1				
	26	tGg	→	tTg		Trp	→	Leu						1
	37	Cag	→	Aag		Gln	→	Lys			1			
	79	Gaa	→	Taa		Glu	→	Stop					1	
	92	gGc	→	gTc		Gly	→	Val					1	
	101	gCc	→	gAc		Ala	→	Asp					1	
	108	agC	→	agA		Ser	→	Arg				2		
	110	cGt	→	cTt	CpG	Arg	→	Leu						1
	115	Ggt	→	Tgt	CpG	Gly	→	Cys				2	1	2^a^
	116	gGt	→	gTt		Gly	→	Val		2^a^				1
	140	gCg	→	gAg	CpG	Ala	→	Glu	1			1	3^a^	1
	143	cGt	→	cTt	CpG	Arg	→	Leu				8^a^	1	
	145	Gaa	→	Taa		Glu	→	Stop	1	1	1			
	185	aGc	→	aTc		Ser	→	Ile	1					1
	186	agC	→	agA		Ser	→	Arg	1					
	189	taC	→	taA	CpG	Tyr	→	Stop			1	3^a^	1	
	208	Gag	→	Tag	CpG	Glu	→	Stop				3	1	
	244	Gaa	→	Taa	CpG	Glu	→	Stop	1	2^a^		2	1	1
	262	Gat	→	Tat		Asp	→	Tyr			1			
	281	gGt	→	gTt		Gly	→	Val					1	
	304	Gaa	→	Taa		Glu	→	Stop	1	1	1			
	320	gCg	→	gAg	CpG	Ala	→	Glu	1					1
	401	tGg	→	tTg		Trp	→	Leu		2^a^				
	402	tgG	→	tgT		Trp	→	Cys		1		1	4^b^	
	406	Gaa	→	Taa		Glu	→	Stop		2^a^	1		3^b^	
	409	Cag	→	Aag		Gln	→	Lys	1					
	413	cCg	→	cAg	CpG	Pro	→	Gln		1		1		
G:C → C:G	6	agC	→	agG	CpG	Ser	→	Arg		1				
	50	cGt	→	cCt	CpG	Arg	→	Pro				1		
	110	cGt	→	cCt	CpG	Arg	→	Pro					1	
	112	Ggc	→	Cgc		Gly	→	Arg	1				1	
	125	cCg	→	cGg	CpG	Pro	→	Arg		1		2^a^		
	127	Ggt	→	Cgt		Gly	→	Arg						1
	130	Gcg	→	Ccg		Ala	→	Pro					1	1
	139	Gcg	→	Ccg		Ala	→	Pro					1	
	186	agC	→	agG		Ser	→	Arg	2	1		1		
	206	cGc	→	cCc	CpG	Arg	→	Pro					1	
	280	Ggt	→	Cgt	CpG	Gly	→	Arg			1	1		
	289	Gcg	→	Ccg		Ala	→	Pro				1		
	290	gCg	→	gGg	CpG	Ala	→	Gly	1					
	340	Gca	→	Cca	CpG	Ala	→	Pro	1	1	1	1	4^b^	1
	413	cCg	→	cGg	CpG	Pro	→	Arg					1	
	418	Gat	→	Cat		Asp	→	His					1	
	442	Cca	→	Gca		Pro	→	Ala	1				1	
A:T → T:A	10	Aaa	→	Taa		Lys	→	Stop		1				
	25	Tgg	→	Agg		Trp	→	Arg					2^a^	1
	44	caT	→	caA		His	→	Gln					1	
	88	Aaa	→	Taa		Lys	→	Stop						2
	146	gAa	→	gTa		Glu	→	Val		3^a^				
	164	gTc	→	gAc		Val	→	Asp			2			
	187	Tac	→	Aac		Tyr	→	Asn	1					
	365	gTt	→	gAt		Val	→	Asp					1	
	375	taT	→	taA		Tyr	→	Stop		1				
	419	gAt	→	gTt		Asp	→	Val			2^a^			
	458	tAa	→	tTa		Stop	→	Leu		1				
	459	taA	→	taT		Stop	→	Tyr			1			
A:T → C:G	1	Atg	→	Ctg		Met	→	Leu			1			
	56	cTc	→	cGc		Leu	→	Arg			1			1
	106	Agc	→	Cgc		Ser	→	Arg		1	1			
	134	tTa	→	tGa		Leu	→	Stop		1				
	312	taT	→	taG		Tyr	→	Stop						1
	419	gAt	→	gCt		Asp	→	Ala		1				1
ᅟ														
Deletion	8–12	gAAAAAt					→	gAAAAt	1	1	1			
−1 base	13	aTa					→	aa		1				
	26–28	tGGGa					→	tGGa					1	1
	34–35	gTTg					→	gTg	1					
	67	aCt					→	at					1	
	86–87	tGGa					→	tGa					1	
	101–102	gCCg					→	gCg					1	
	114	gCg					→	gg			1			
	124–125	aCCg					→	aCg						1
	126–128	cGGGt					→	cGGt		1			1	
	129	gTg					→	gg		1				
	170–171	aCCg					→	aCg			1		2^a^	
	176	tGt					→	tt						1
	198	aCa					→	aa				1		
	217	aGt					→	at					1	
	244	cGa					→	ca				1		
	247–248	aGGc					→	aGc						1
	249	gCt					→	gt			1			
-	266	gAc					→	gc			1			
	270–271	tGGt					→	tGt		2^a^		1		
	278–279	aCCg					→	aCg					1	
	285	gTa					→	ga					1	
	293–294	gTTg					→	gTg					1	
	315–318	cAAAAg					→	cAAAg					1	
	319	aGc					→	ac		1			1	
	332–333	aCCa					→	aCa		1				
	401–402	tGGa					→	tGa			1			
	416–418	tGGGa					→	tGGa	1	1			1	
	442–443	gCCa					→	gCa				1		
>2	107–109	aGCCg					→	ag					2	
	114–120	gCGGTCTGg					→	gg						1
	129–139	gTGCGTTACTGGc					→	gc			2			
	140–152	gCGCGTGAACTGGGt					→	gt		1				
	161–442									1				
	243–248	gCGAAGGc					→	gc						1
	300–306	tTCGTGAAa					→	ta						1
	375–380	aTGTTGTt					→	at			2			
Insertion	25	cTg					→	cTTg					1	
	75	ct					→	cAt		3^a^				
	124	ac					→	aTc					2^a^	
	136	aCt					→	aCCt					1	
	223–225	gAAAc					→	gAAAAc		1				
	229	cGc					→	cGGc						1
	286	gt					→	gATACCGGTGGt		1				
	335–337	aTCTt					→	aTCTTCTt						1
	362	cTg					→	cTTg					1	
	390	cc					→	cTc					1	
	392–393	cAAg					→	cAAAg						1
	401–402	tGGa					→	tGGGa			1	2		
Other	26–27	tGGg					→	tTg				1		
	59–60	gCAa					→	gGa					1	
	100–102	tGCCg					→	tTg					1	
	140–141	gCGc					→	gAAc				1		
	304	tGa					→	tAAa		1				
Total									38	92	43	42	79	49

^a, b, and c^Mutations found in 2, 3, and 4 different mice, respectively

^d^Combined data of our previous studies [11, 17, 18]

^e^Our previous data [11]

CpG: mutation at CpG site

Our results showed that the predominant type of mutation in the lungs of the vehicle control *gpt* delta mice was G:C to A:T transitions in all age groups; these transitions have also been shown to be the predominant type of mutation in liver and other organs in both newborn and 23-month–old *lacZ*-transgenic mouse [19]. G:C to A:T transitions are recognized to be more frequently induced on CpG sites, in which cytosines tend to be methylated, by spontaneous deamination of methylated cytosines to form thymine residues, resulting in the formation of G:T mispairs [25]. Most cytosines in CpG sites in the liver of *gpt* gene integrated in the genomic DNA are highly methylated [26], and are therefore hotspots of spontaneous mutation in the control mice, such as G:C to A:T transitions at nucleotides 64, 110, and 115 are at CpG sites (Table 3). Methyl-CpG Domain Protein 4 (MBD4) and thymine-DNA glycosylase (TDG) [27] are mismatch repair enzymes that correct G:T mispairs by excising the mispaired thymine [28]. We consider that decrease in these DNA glycosylases and other mismatch repair enzymes possibly contribute to the observed increase in occurrence of G:C to A:T transitions in aged animals [19, 21, 29].

In B[a]P-instilled lung of *gpt* delta mice, G:C to A:T transitions were shown to increase in an age-dependent manner (Table 2); this type of mutation was most predominant in 24-month–old mice. In contrast, G:C to T:A transversions (a landmark mutation of B[a]P-DNA adduct formation possibly induced by translesional DNA synthesis [30]) were the major base substitution in B[a]P-instilled lungs of 3-month–old mice. As summarized in Table 4, estimation of specific mutant frequency ([Average mutant frequency in Table 1] × [% mutant of G:C to A:T transition or G:C to T:A transversion of corresponding group in Table 2]) showed that G:C to T:A transversion was markedly increased in B[a]P-instilled lung of 3-month–old mice (1.39 × 10^−5^) compared to the age-matched control (0.12 × 10^−5^) but the increase of G:C to A:T transition by B[a]P instillation was not observed in 3-month–old mice. On the other hand, in B[a]P-instilled lung of 24-month–old mice, specific mutant frequency of G:C to A:T transition (0.92 × 10^−5^) was elevated significantly (*P* < 0.01, Chi-square test) compared to the age-matched control (0.35 × 10^−5^), while G:C to T:A transversion was also elevated by B[a]P instillation. These observations suggest that G:C to T:A transversion was a predominant mutation for elevation of mutant frequency in B[a]P-instilled lung of 3-month–old mice, but in 24-month–old mice, induction of G:C to A:T transition as well as G:C to T:A transversion drove the elevation of mutant frequency by B[a]P instillation. Elevated levels of metabolic activation in young animals [22] might accelerate the induction of G:C to T:A transversions.

**Table 4:** Specific mutant frequency of G:C to A:T transition and G:C to T:A transversion on *gpt* gene from the lung of B[a]P-instilled and control mice

Type of mutation in the *gpt* gene	Control (months)			B[*a*]P (months)		
	3	11	24	3	11	24
	Specific mutant frequency* (×10^−5^)					
G:C → A:T	0.27	0.44	0.35	0.25	0.42	0.92**
G:C → T:A	0.12	0.18	0.14	1.39	0.40	0.41

*Specific mutant frequency = [Average mutant frequency in Table 1] × [% mutant of G:C to A:T transition or G:C to T:A transversion of corresponding group in Table 2]

**P < 0.001 (Chi-square test) for comparison between B[*a*]P-instilled mice and age-matched control mice

Our mutation spectrum analysis of B[a]P-instilled lungs revealed that not only the overall percentage of G:C to A:T transitions but the percentage of G:C to A:T transitions at non-CpG sites increased with age (8 %, 12 %, and 25 % [‘the percentage of total G:C to A:T transitions’ minus ‘the percentage of total G:C to A:T transitions at CpG sites’] at 3 months, 11 months, and 24 months, respectively). A possible mechanism for the induction of G:C to A:T transitions in B[a]P-instilled old mice is that spontaneous deamination of cytosine at CpG sites was elevated in the lungs of these mice by instillation of B[a]P, resulting in an increase in the percentage of G:C to A:T transitions at CpG sites in 24-month–old mice (16 %). A decrease in mismatch repair [29] might also contribute to the increase in occurrence of G:C to A:T transitions in B[a]P-instilled aged animals. Another possibility is that DNA cytidine deaminase is activated in the lungs of aged mice by instillation of B[a]P. This enzyme catalyzes the conversion of cytosine to uracil at both CpG and non-CpG sites, which leads to G:U mispair formation and hence mutation of G:C to A:T. The hypermutation induced by DNA cytidine deaminase plays a role in creating antibody diversity in the variable regions [31], and expression of this enzyme causes genomic instability related to cancer and other diseases [32]. We speculate that B[a]P induces DNA cytidine deaminase in the lungs of aged animals resulting in the induction of G:C to A:T transversions at non-CpG sites.

The biological significance of the age-related increase in G:C to A:T transitions in both B[a]P-instilled and control mice remains unclear. G:C to A:T transitions are induced on codons 12 and 13 of the K-ras gene in lung tumors spontaneously induced in either p53-suppressed old mice or age-matched wild-type mice (13- to 24-month–old mice) [33]. Recently, G:C to A:T transitions were shown to occur frequently in six types of human tumors (lung adenocarcinoma, lung squamous cell carcinoma], bladder, cervix, head and neck), in which APOBEC3B, an isoform of DNA-cytidine deaminase, was upregulated [34], and lung adenocarcinoma developed in transgenic mice with constitutive expression of DNA-cytidine deaminase [35]. These observations suggest that an increase in G:C to A:T transitions may contribute to not only spontaneous but also B[a]P-induced tumorigenesis in the lungs of aged mice. However, further studies are required to reveal the mechanism that G:C to A:T transitions induced in the lung cause cancer and other diseases in the old age.

## Conclusions

Our observations indicate that the order of the age groups in terms of highest to lowest fold of increase in mutant frequency in the lungs following instillation of B[a]P was 3-, 24-, and 11-month–old mice, suggesting that young mice are the age-group most susceptible to B[a]P. G:C to T:A transversion was shown to be a predominant mutation for elevation of mutant frequency in B[a]P-instilled lung of 3-month–old mice, but in 24-month–old mice, induction of G:C to A:T transition as well as G:C to T:A transversion drove the elevation of mutant frequency by B[a]P instillation. We speculate that B[a]P induces DNA cytidine deaminase in the lungs of aged animals resulting in the induction of G:C to A:T transversions at non-CpG sites.
